# Exponential random graph model parameter estimation for very large directed networks

**DOI:** 10.1371/journal.pone.0227804

**Published:** 2020-01-24

**Authors:** Alex Stivala, Garry Robins, Alessandro Lomi

**Affiliations:** 1 Institute of Computational Science, Università della Svizzera italiana, Lugano, Ticino, Switzerland; 2 Centre for Transformative Innovation, Swinburne University of Technology, Melbourne, Victoria, Australia; 3 Melbourne School of Psychological Sciences, The University of Melbourne, Melbourne, Victoria, Australia; 4 The University of Exeter Business School, The University of Exeter, Exeter, Devon, United Kingdom; Universidad Rey Juan Carlos, SPAIN

## Abstract

Exponential random graph models (ERGMs) are widely used for modeling social networks observed at one point in time. However the computational difficulty of ERGM parameter estimation has limited the practical application of this class of models to relatively small networks, up to a few thousand nodes at most, with usually only a few hundred nodes or fewer. In the case of undirected networks, snowball sampling can be used to find ERGM parameter estimates of larger networks via network samples, and recently published improvements in ERGM network distribution sampling and ERGM estimation algorithms have allowed ERGM parameter estimates of undirected networks with over one hundred thousand nodes to be made. However the implementations of these algorithms to date have been limited in their scalability, and also restricted to undirected networks. Here we describe an implementation of the recently published Equilibrium Expectation (EE) algorithm for ERGM parameter estimation of large directed networks. We test it on some simulated networks, and demonstrate its application to an online social network with over 1.6 million nodes.

## Introduction

Exponential random graph models (ERGMs) are a class of statistical model often used for modeling social networks [1, 2]. Parameter estimation in these models is a computationally difficult problem, and algorithms based on Markov chain Monte Carlo (MCMC) are generally used [2–10]. The computational time required by these methods places a limit on the size of networks for which models can be estimated in practice. A recently published new algorithm for sampling from the ERGM distribution can reduce this time by an order of magnitude [11], and a new estimation algorithm even more [12], however scalability is still a problem when extremely large networks are considered. It is also worth noting that the state space for a directed network is far larger than for an undirected network with the same number of nodes [13], and so this problem is even more difficult in the case of directed networks.

One solution to this problem is to take snowball samples [14–18] from the original network, and estimate ERGM parameters from these [19, 20]. The first description of such a method was [19]. However this method requires that estimation over the entire set of random tie variables is feasible, limiting the size of networks to which the method can be applied in practice. A more recently proposed method [20] is to estimate parameters for each sample in parallel with conditional estimation [21], combining the estimates with a meta-analysis [22] or using bootstrap methods [23] to estimate the standard errors. This work, however, was only applied to undirected networks.

However the problem of directly estimating ERGM parameters for a very large network (rather than from snowball samples) remains, particularly for directed networks where snowball sampling is not straightforward. Here we describe an implementation of the Equilibrium Expectation (EE) method [12] extended to directed networks, which is scalable and efficient enough to be used to estimate ERGM parameters for networks with over one million nodes.

Hunter & Handcock (p. 581 of [6]) note that the largest network estimated to date (in 2006) was the *N* = 2209 nodes adolescent friendship network estimated by Hunter, Goodreau, & Handcock [24]. However this network was treated as undirected. Larger undirected networks subsequently had ERGM models estimated indirectly by snowball sampling, with the largest having 40 421 nodes [20]. By using an improved ERGM distribution sampler, Byshkin et al. [11] could directly estimate ERGM parameters for a 3061 node patient transfer network (treated as undirected), and the Equilibrium Expectation algorithm was demonstrated on an undirected online social network with 104 103 nodes [12]. Using the implementation described in this paper, modified to use a simplified EE algorithm, Borisenko, Byshkin, & Lomi [25] are able to estimate a simple ERGM model of a 75 879 node directed network.

We note that social networks are typically sparse, and we assume sparsity for efficient data structures. There is some specific work on sampling methods for ERGMs for large sufficiently dense networks with additional assumptions [26] such as the presence of block structure [27, 28] but here we assume only sparsity, and that the network can plausibly be described by an exponential random graph model.

In this paper, we describe an implementation of the EE algorithm, including the improved fixed density ERGM sampler [11] for application to directed networks. By implementing these algorithms and the associated computations of change statistics in a more efficient and scalable manner, we are able to estimate ERGM parameters for networks far larger than previously possible, even using existing implementations of the algorithms used for the computational experiments in the papers originally describing them [11, 12]. The implementation we describe allows ERGM parameter estimation for a model of a directed network with over one million nodes, while existing methods are only practical on networks of a few thousand nodes at most. We test the implementation first on simulated networks with known model parameters, in order to validate that it works correctly, and then demonstrate its application to an online social network with over 1.6 million nodes.

### Exponential random graph models

An ERGM is a probability distribution with the form

$$\begin{array}{c} \text{Pr}\left(X=x\right)=\frac{1}{\kappa }\;\text{exp}\;(\sum_{A}\theta_{A}z_{A}\left(x\right)) \end{array}$$ (1)

where

- *X* = [*X*_*ij*_] is a 0-1 matrix of random tie variables,
- *x* is a realization of *X*,
- *A* is a *configuration*, a (small) set of nodes and a subset of ties between them,
- *z*_*A*_(*x*) is the network statistic for configuration *A*,
- *θ*_*A*_ is a model parameter corresponding to configuration *A*,
- *κ* is a normalizing constant to ensure a proper distribution.

Which configurations *A* are allowed depends on the assumptions as to which ties are independent. Here we will use the *social circuit dependence* assumption [7, 29], under which two potential ties are conditionally dependent exactly when, if they were observed, they would form a 4-cycle in the network [13]. Configurations allowed by other, simpler, dependence assumptions (Bernoulli, dyad-independent, Markov (pp. 56–57 of [1]) are also allowed in these models.

Under this assumption, we will now describe the structural configurations used in this work. In the following, *N* is the number of nodes in the network.

The simplest configuration, included in every model, is Arc, analogous to the intercept in a regression. Arc is included to account for the overall density of the network observed. Its corresponding statistic is $z_{L}=\sum_{i=1}^{N}\sum_{j=1}^{N}x_{ij}$, the number of arcs in the graph. The Reciprocity parameter is used to test for propensity of arcs to be reciprocated, and its statistic is $z_{\text{Reciprocity}}=\sum_{i=1}^{N}\sum_{j=1}^{N}x_{ij}x_{ji}$.

**Fig 1:**
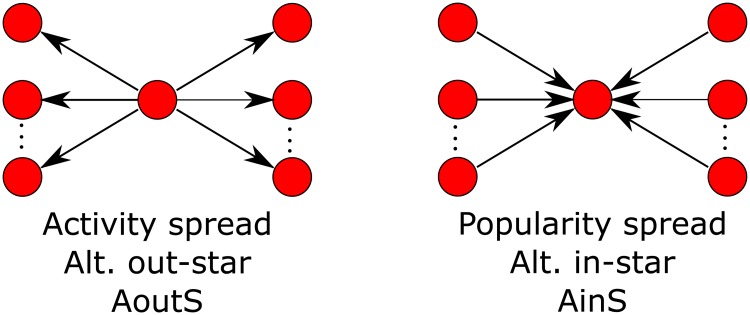
Alternating *k*-star structures for modeling degree distribution in directed networks. Alternating *k*-in-star models popularity spread and alternating *k*-out-star models activity spread.

The degree distribution in a directed network is modeled with the alternating *k*-out-star and alternating *k*-in-star configurations defined by [29] and illustrated in Fig 1. The statistic for *k*-out-star is defined as:

$$\begin{array}{c} z_{\text{AoutS}}=\sum_{k=2}^{N-1}{\left(-1\right)}^{k}\frac{S_{k}^{Out}}{\lambda^{k-2}} \end{array}$$ (2)

where $S_{k}^{Out}$ is the number of *k*-out-stars and λ ≥ 1 is a damping parameter. We use λ = 2 in this work, as used previously in, for example, [20, 29]. We note that in a more general form of ERGM, the curved exponential family random graph model [6], it is also possible to estimate (a parameter equivalent to) the parameter λ, and this is routinely done using the statnet software [30–33]. However the EE algorithm requires that every model parameter has a corresponding change statistic, and so cannot estimate curved ERGMs [12]. For this reason assume a fixed value for λ.

The *z*_AinS_ statistic for *k*-in-stars is defined similarly.

**Fig 2:**
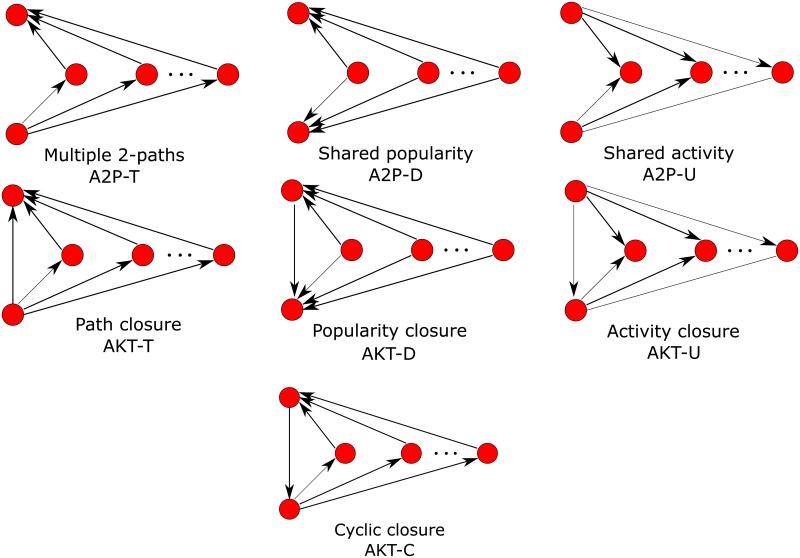
Alternating transitive *k*-triangle and alternating *k*-2-paths structures. These are used for modeling social circuit dependence including path closure and shared popularity. The A2P-TD configuration counts *k*-2-paths (A2P-T) and shared popularity (A2P-D) configurations in a single configuration, adjusting for double counting.

Path closure and multiple connectivity are modeled with the alternating transitive *k*-triangles and alternating two-paths effects defined by [29] and illustrated in Fig 2. These statistics are defined as [34]:

$$\begin{array}{c} z_{\text{AT}-T}=\lambda \sum_{i=1}^{N}\sum_{j=1}^{N}x_{ij}[1-(1-\frac{1}{\lambda }{)}^{L_{2}\left(i,j\right)}] \end{array}$$ (3)

where $L_{2}\left(i,j\right)=\sum_{h=1}^{N}x_{ih}x_{hj}$ is the number of directed two-paths from *i* to *j*, and

$$\begin{array}{c} z_{A2P-T}=\lambda \sum_{i<j}^{N}[1-(1-\frac{1}{\lambda }{)}^{L_{2}\left(i,j\right)}] \end{array}$$ (4)

As well as path closure (AT-T) we can also define cyclic closure (AT-C), in which arcs constituting the triangles form a cycle. Its statistic *z*_AT−C_ is defined analogously to *z*_AT−T_ but counting cyclic *k*-triangles rather than transitive *k*-triangles:

$$\begin{array}{c} z_{\text{AT}-C}=\lambda \sum_{i=1}^{N}\sum_{j=1}^{N}x_{ji}[1-(1-\frac{1}{\lambda }{)}^{L_{2}\left(i,j\right)}] \end{array}$$ (5)

We also include the *shared popularity* configuration A2P-D [13], the statistic for which *z*_A2P−D_ is defined analogously to *z*_A2P−T_, but rather than counting directed paths between two nodes via *k* intermediate nodes, it counts “paths” where the each of the *k* intermediate nodes have arcs directed towards each of the two nodes (see Fig 2):

$$\begin{array}{c} z_{A2P-D}=\lambda \sum_{i<j}^{N}[1-(1-\frac{1}{\lambda }{)}^{L_{2D}\left(i,j\right)}] \end{array}$$ (6)

where $L_{2D}\left(i,j\right)=\sum_{h=1}^{N}x_{hi}x_{hj}$. We then define the configuration A2P-TD which is the sum of the A2P-D and A2P-T statistics, adjusting for double-counting:

$$\begin{array}{c} z_{A2P-\text{TD}}=z_{A2P-T}+\frac{z_{A2P-D}}{2} \end{array}$$ (7)

The *shared activity* configuration A2P-U [13], the statistic for which is *z*_A2P−U_ is similar to A2P-D, but counts “paths” where each of the *k* intermediate nodes have arcs directed from the pairs of nodes to the intermediate nodes (see Fig 2):

$$\begin{array}{c} z_{A2P-U}=\lambda \sum_{i<j}^{N}[1-(1-\frac{1}{\lambda }{)}^{L_{2U}\left(i,j\right)}] \end{array}$$ (8)

where $L_{2U}\left(i,j\right)=\sum_{h=1}^{N}x_{ih}x_{jh}$.

The statistics for the closures corresponding to the open path types A2P-D and A2P-U, popularity closure (AKT-D) and activity closure (AKT-U), respectively, are defined similarly to the way path closure (AKT-T) is defined for the corresponding multiple two-paths A2P-T.

These configurations are illustrated in Fig 2.

**Fig 3:**
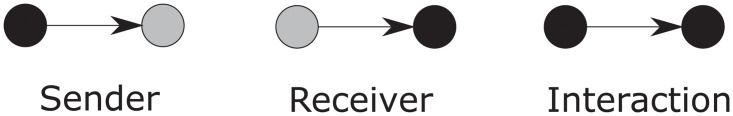
Binary attribute configurations. The dark nodes represent actors with the binary attribute, and the lighter shaded nodes represent actors with or without the attribute.

In addition, we will allow nodes to have binary, categorical, or continuous attributes, and use the following additional configurations using these nodal attributes. For the binary attribute, we use the four configurations *Sender*, *Receiver*, and *Interaction*, illustrated in Fig 3. The Sender parameter indicates increased propensity of a node with the True value of the binary attribute to “send” a tie to another node, and the Receiver the increased propensity of a node with the True value of the attribute to “receive” a tie from another node (both irrespective of the attribute value of the other node). The Interaction parameter indicates increased propensity for two nodes both with the True value of the attribute to have an arc connecting them. The corresponding statistics are defined as follows (where we now use the notation ∑_*i*,*j*_ for summation over all pairs of nodes *i* ∈ {1…*N*}, *j* ∈ {1…*N*}, *i* ≠ *j*):

$$z_{\text{Sender}}=\sum_{i,j}a_{i}x_{ij}$$ (9)

$$z_{\text{Receiver}}=\sum_{i,j}a_{j}x_{ij}$$ (10)

$$z_{\text{Interaction}}=\sum_{i,j}a_{i}a_{j}x_{ij}$$ (11)

where *a*_*i*_ ∈ {0, 1} is the value of the binary attribute on node *i*.

**Fig 4:**
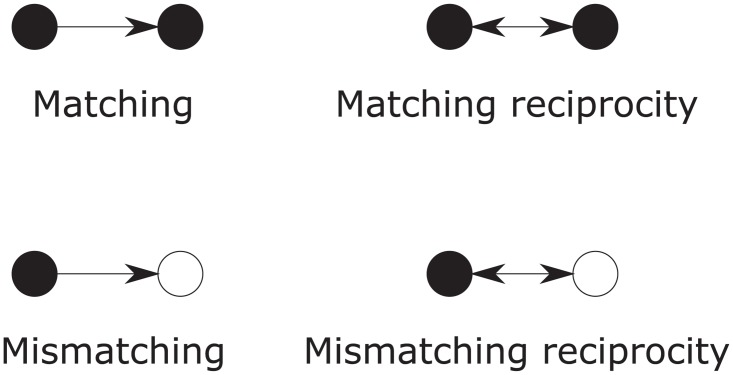
Categorical attribute configurations. The filled and empty nodes represent actors with two different values of the categorical attribute.

For the categorical attribute, the *Matching* and *Mismatching* parameters indicate the increased propensity for a node to send a tie to another node with, respectively, the same, or different, value of the categorical attribute. The *Matching reciprocity* and *Mismatching reciprocity* parameters indicate the increased propensity for such ties to be reciprocated. These configurations are illustrated in Fig 4 and the corresponding statistics are defined by:

$$z_{\text{Matching}}=\sum_{i,j}\delta_{c_{i},c_{j}}x_{ij}$$ (12)

$$z_{\text{Mismatching}}=\sum_{i,j}\left(1-\delta_{c_{i},c_{j}}\right)x_{ij}$$ (13)

$$z_{\text{Matching}\;\text{reciprocity}}=\sum_{i,j}\delta_{c_{i},c_{j}}x_{ij}x_{ji}$$ (14)

$$z_{\text{Mismatching}\;\text{reciprocity}}=\sum_{i,j}\left(1-\delta_{c_{i},c_{j}}\right)x_{ij}x_{ji}$$ (15)

where *c*_*i*_ is the value of the categorical attribute at node *i* and *δ*_*x*,*y*_ is the Kronecker delta function

$$\begin{array}{c} \delta_{x,y}=\{\begin{array}{ccc} 0 & \text{if} & x\neq y,\\ 1 & \text{if} & x=y. \end{array} \end{array}$$ (16)

For a continuous attribute *u*_*i*_ on a node *i*, we also define the (continuous) Sender, Receiver and Difference statistics as follows:

$$z_{\text{continuousSender}}=\sum_{i,j}u_{i}x_{ij}$$ (17)

$$z_{\text{continuousReceiver}}=\sum_{i,j}u_{j}x_{ij}$$ (18)

$$z_{\text{Diff}}=\sum_{i,j}\left|u_{i}-u_{j}\right|x_{ij}$$ (19)

These indicate, respectively, the increased propensity of a node to send ties for higher values of its continuous attribute, the increased propensity of a node to receive ties for higher values of its continuous attribute, and the increased propensity of nodes to have a tie between them for smaller absolute differences in their continuous attributes. The latter is a simple measure of homophily, the tendency for nodes with similar values of the attribute to have a tie between them.

Note that in ERGM estimation algorithms these statistics as defined above never actually need to be computed directly. Instead only the corresponding *change statistics* are computed [6, 10, 24, 29, 31]. The change statistic is the change in the statistic due to the addition or deletion of an arc, which is much faster to compute. For example the most basic statistic is *z*_*L*_, the count of the number of arcs in the graph. Computing this statistic therefore requires counting the number of arcs in the graph, however the corresponding change statistic is simply the constant 1 (or -1 for deleting an arc): adding an arc increases the statistic by 1, and deleting an arc decreases it by 1.

### Equilibrium expectation algorithm

Monte Carlo based methods, such as Markov chain Monte Carlo maximum likelihood estimation (MCMCMLE) [6] and stochastic approximation [5] as well as Bayesian methods [8], as reviewed for example by Hunter et al. [10], all require drawing simulated networks from the ERGM distribution. This can be achieved using a Metropolis–Hastings algorithm, and a number of samplers are available [5, 10, 11, 35]. However all these methods require that a number of network samples are drawn from the stationary ERGM distribution, for each updated value of the parameter vector being estimated, which may require a very large number of iterations, and limits the size of networks to which these methods can be applied in practice.

In contrast, the EE algorithm [12] does not require these potentially very long MCMC simulations between parameter updates. The EE algorithm is related to persistent contrastive divergence (PCD) [25, 36, 37] and is fast because it adjusts its parameters according the difference between the observed network statistics and the statistics of a current non-equilibrium state of the Markov chain of simulated networks. It may be thought of as a kind of gradient ascent method, and depends on the property of the exponential family (to which the ERGM distribution belongs) that the expected value of a statistic is a monotonically increasing function of the corresponding parameter (see Ch. 8 of [38]). It works by starting the chain of simulated networks at the observed network (not the empty network for example), and taking only a small number of Metropolis–Hastings steps, before adjusting the estimated parameter values according to the divergence of the simulated network statistics from the observed network statistics. After sufficiently many iterations of this process (which in practice is many orders of magnitude smaller than the number of Metropolis–Hastings steps required to find the stationary ERGM distribution), the divergence of each of the statistics from the observed statistics oscillates around zero, and the corresponding parameters oscillate around a value which is taken to be an estimate of the MLE.

A version of contrastive divergence (CD) [39] is used to compute initial values of the ERGM parameter estimates [40, 41] for the EE algorithm [12]. Details of the EE algorithm, as first described in the Supplementary Information of [12], and of the IFD sampler, [11] are provided in S1 Appendix.

## Materials and methods

### Parameter estimation

Parameters are estimated using a new implementation of the EE algorithm, which we call EstimNetDirected. This implementation has change statistics for directed (rather than undirected as in the original description [12]) networks, and uses efficient data structures in order to scale to very large (over one million node) networks. Both the “basic” ERGM sampler (as used in the PNet [42] software) and the improved fixed density (IFD) ERGM sampler [11] are implemented.

The network is stored as an adjacency list data structure for space efficiency and fast computation of change statistics. A “reversed” adjacency list is also maintained. This stores, for each node *j*, a list of nodes *i* for which the arc *i* → *j* exists. This allows efficient computation of change statistics that require the in-neighbours of a node. In addition, a flat list of all arcs is maintained, for efficient implementation of the IFD sampler, which requires finding an arc uniformly at random for arc deletion moves [11].

For efficient computation of the “alternating” two-path and triangle change statistics, it is necessary to keep track of the counts of two-paths between each pair of nodes. It is not scalable to store these two-paths matrices as arrays as in earlier implementations [12, 42], so instead hash tables can be used, where the key is a node pair (*i*, *j*) and the value is the relevant two-path count (which is zero if the key is not present). This takes advantage of the sparsity (approximately 0.06% nonzero in the empirical example described here) of these matrices and still allows fast (asymptotically constant time) lookup. In addition, a Bloom filter [43] is used so that the overwhelmingly more frequent case of looking up an entry that is not present is faster. During the MCMC ERGM sampling process, in which arcs are added and deleted, entries in the two-path tables that fall to zero are deleted, in order to stop the tables from growing in size indefinitely, however this diminishes the effectiveness of the Bloom filter.

We run a number of estimations independently (and in parallel to minimize elapsed time).

### Standard error estimation

For each parallel estimation run, the point estimates and their standard errors are estimated. The point estimate (mean) and asymptotic covariance matrix for MCMC standard error are estimated using the multivariate batch means method [44, 45] using the mcmcse R package [46]. The covariance matrix for the error in inherent in using the MLE is estimated as the inverse of the covariance matrix of the simulated statistics (Fisher information matrix) [5, 6] also using mcmcse. The total estimated covariance matrix is then estimated as the sum of these two covariance matrices, and from this we compute the standard error as the square root of the diagonal.

The overall estimate and its standard error are then computed as the inverse variance weighted average (see Ch. 4 of [47]) of the results calculated from each independent (parallel) estimation.

### Implementation and availability

EstimNetDirected is implemented in the C programming language using the message passing interface (MPI) for parallelization on computing clusters. The uthash [48] macro collection is used to implement hash tables (including Bloom filter) and the Random123 counter-based pseudo-random number generator [49] is used to generate pseudo-random numbers for the MCMC process. Scripts for processing network data formats, estimating standard errors, and generating plots and fitting heavy-tailed distributions are written in R and Python and use the igraph [50], SNAP [51], ggplot2 [52], PropCIs [53], mcmcse [46], and poweRlaw [54] packages.

All source code and scripts are publicly available on GitHub at https://github.com/stivalaa/EstimNetDirected.

### Simulated networks

To ensure that the parameter estimation algorithm works correctly, we first apply it to estimating ERGM parameters of networks with known true values, and measure the bias, root mean square error (RMSE), coverage, and Type I and Type II error rates in statistical inference. To do so we simulate sets of 100 networks sampled from an ERGM network distribution with known parameters, and then estimate the parameters of each of the 100 networks with EstimNetDirected. This allows the mean bias and RMSE to be estimated. The coverage is then the percentage of the 95% confidence intervals which contain the true value of the parameter. Coverage higher than the nominal 95% indicates overly conservative (high) estimates of the standard error, and coverage lower than the nominal value indicates overly optimistic (low) values of the standard error (uncertainty). In addition, we estimate the Type II error rate in inference (the false negative rate), as the percentage of estimations in which the estimated 95% confidence interval includes zero.

To estimate the Type I error rate (false positive rate) for inference of an ERGM parameter significance, we generate simulated networks in which the parameter in question is zero, and proceed as just described. Then the Type I error rate is estimated as the percentage of estimations in which the 95% confidence interval does not include zero.

We generate two sets of graphs from ERGM distributions, both with *N* = 2000 nodes. First, a network with binary node attributes and parameters (Arc, Reciprocity, AinS, AoutS, AT-T, A2P-TD, Interaction, Sender, Receiver) = (-1.00, 4.25, -2.00, -1.50, 0.60, -0.15, 2.00, 1.50, 1.00), and, second, a network with categorical node attributes and parameters (Arc, Reciprocity, AinS, AoutS, AT-T, A2P-TD, Matching, Matching reciprocity) = (-1.00, 4.25, -2.00, -1.50, 1.00, -0.15, 1.50, 2.00). For each of the two sets of parameters, we generated 100 samples from a network distribution with those parameters using PNet [42]. For networks with a binary attribute, 50 of the nodes (2.5%), selected at random, have the True value, and the rest False. For networks with a categorical attribute, the attribute at each node is assigned one of three possible values uniformly at random. The networks are sampled with sufficient burn-in (of the order of 10^9^ iterations) to ensure initialization effects are minimized, and samples are taken sufficiently far apart (separation of the order of 10^8^ iterations) to ensure that they are essentially independent. Table 1 shows summary statistics of the simulated networks.

**Table 1:** Statistics of the simulated directed networks.

N	Attributes	Zero effect	Mean components	Mean degree	Mean density	Mean c.c.
2000	Binary	None	1.05	2.65	0.00132	0.00482
2000	Categorical	None	1.00	3.79	0.00189	0.05655
2000	Binary	Sender	1.02	2.60	0.00130	0.00323
2000	Binary	Receiver	1.03	2.61	0.00131	0.00338
2000	Binary	Reciprocity	1.03	2.58	0.00129	0.00339
2000	Binary	Interaction	1.00	2.63	0.00131	0.00326
2000	Binary	AT-T	1.00	2.63	0.00132	0.00215
2000	Binary	A2P-TD	1.00	3.68	0.00184	0.13192
2000	Binary	AinS	1.00	9.95	0.00498	0.32332
2000	Binary	AoutS	1.00	6.74	0.00337	0.36730
2000	Categorical	Matching	1.04	2.67	0.00134	0.00643
2000	Categorical	Match. Recip.	1.00	3.16	0.00158	0.00966
2000	Categorical	Reciprocity	1.00	3.04	0.00152	0.00707
2000	Categorical	AT-T	1.00	3.49	0.00175	0.00331
2000	Categorical	A2P-TD	5.97	1.83	0.00092	0.00802
2000	Categorical	AinS	1.00	5.38	0.00269	0.10851
2000	Categorical	AoutS	1.11	3.16	0.00158	0.04159

“c.c.” is the global clustering coefficient. Note that for the Categorical attribute networks with AinS, AoutS, and A2P zero effects, the Arc parameter is set to -4.0 rather than -1.0 to avoid the network becoming dense.

### Empirical network

As an example application we use EstimNetDirected to estimate an ERGM model of the Pokec online social network [55] which is publicly available from the Stanford large network dataset collection [56]. Pokec is the most popular online social network in Slovakia [55] and represents a sizable percentage of Slovakia’s population [57]. This publicly available data is also unusual and particularly useful as a test case for social network algorithms as it is the entire online social network at a point in time, rather than a sample as is often the case, and the nodes are annotated with attributes, specifically including gender, age, and region (187 of them, Slovakia or elsewhere) which we use as nodal covariates in the model.

This network has 1 632 803 nodes and 30 622 564 arcs (directed graph density approximately 10^−5^ and mean degree 37.5). The nodes are users of the Pokec online social network, and arcs represent directed “friendship” relations, i.e. unlike many online social networks, “friendships” are not assumed to be automatically reciprocated (undirected). In fact only approximately 54% of the “friendship” relations are reciprocated. More details and descriptive statistics of this network are in [55].

The Pokec online social network has been previously used as a test bed for social network analysis algorithms, and in particular by Kleineberg & Boguñá [57] who use it to test a model of the evolution of an online social network. They treat the final state of the network as a representation of a true social network, as do we, in order to demonstrate EstimNetDirected estimation of an ERGM, which is a cross-sectional network model. However they treat the network as undirected, including only reciprocated ties, while we maintain the directed nature of the network.

**Fig 5:**
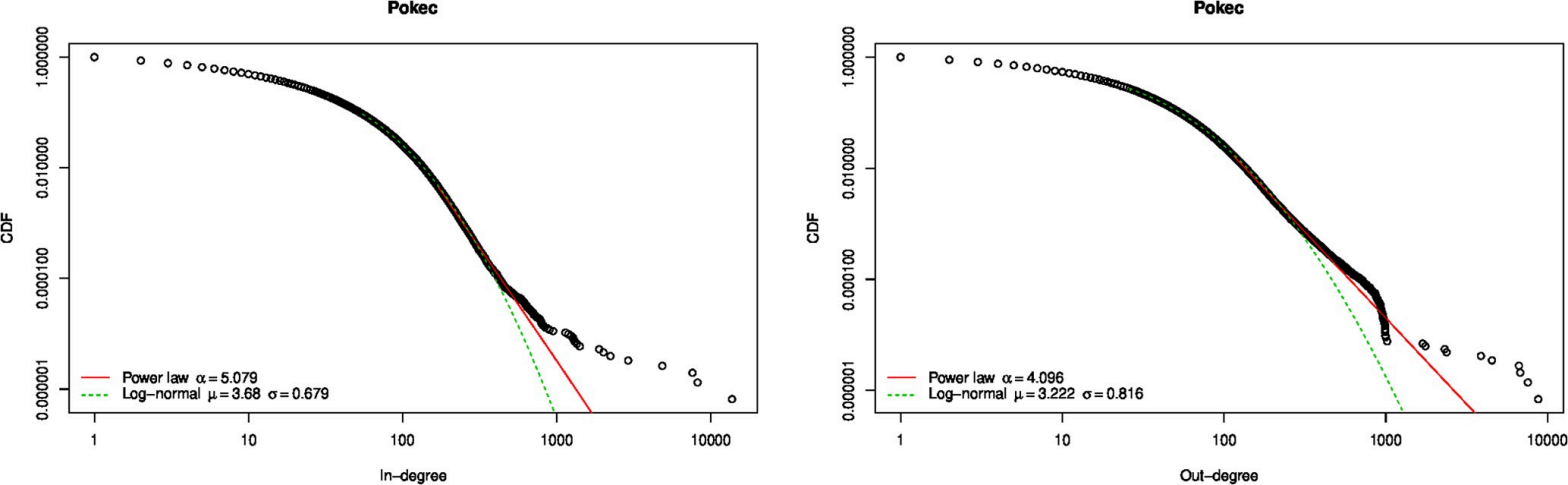
Pokec network degree distribution. Neither the in- nor the out-degree distribution are consistent with power law or log-normal distributions (*p* < 0.01).

The Pokec degree distribution was described as “scale-free” in [55], but based only on visual examination of the degree-frequency plot. This technique, or similarly, fitting a straight line to a degree-frequency log-log plot, is now well known to be not a sound technique for assessing whether a distribution is scale-free or follows a power law [58–60]. Using the statistical method described by Clauset et al. [58] as implemented in the powerRlaw R package [54], we find that the neither the in- nor out-degree distribution of the Pokec online social network is consistent with a power law distribution (Fig 5).

Nevertheless, it is clear from Fig 5 that there are *hubs* in the network, that is, nodes with an order of magnitude higher degree than most other nodes. In particular, there is a noticeable “break” in the empirical cumulative distribution function (CDF) plot at degree 1000, most noticeable for the out-degree distribution. According to Takac & Zabovsky, “hubs in Pokec are not people but commercial companies…” (p. 5 of [55]).

**Fig 6:**
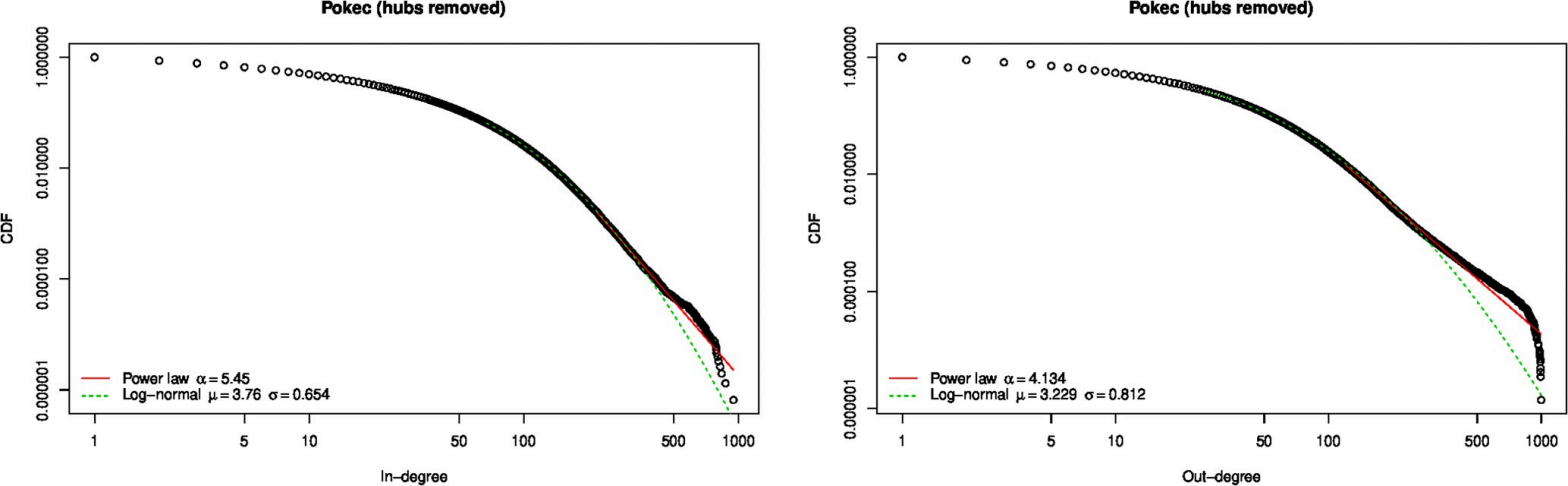
Pokec network degree distribution after hub nodes are removed. After removing the 20 nodes that have in- or out-degree greater than 1000, the resulting network’s in-degree distribution is consistent with a power law distribution, but not with a log-normal distribution (*p* < 0.01). The out-degree distribution is consistent with neither a power law nor a log-normal distribution (*p* < 0.01).

Based on these observations, and on the fact that an initial attempts to estimate an ERGM for the entire network did not converge, we remove all nodes with in- or out-degree greater than 1000 from the network. There are only 20 such nodes (0.001% of the nodes), and removing them does not significantly change the density or mean degree. However it breaks the network, which was initially a single connected component, into 577 components, although the giant component has 1 632 199 nodes (99.96% of the total). This indicates that these hubs are not performing the function of holding different components of the network together into a connected whole, as their removal only results in the creation of a relatively small number of isolated nodes, rather than splitting the network into multiple large components, and that therefore their removal is not substantively changing the nature of the network structure. The in-degree distribution of network with hubs removed is consistent with a power law, although the out-degree distribution is not (Fig 6).

The EstimNetDirected parameter settings used in the estimations are detailed in Table A in S1 Appendix.

### Convergence tests

As in [12] we use a t-ratio check for convergence, but for larger directed networks we weaken the criterion to conclude non-convergence if the absolute value of any parameter’s t-ratio is greater than 0.3. If the covariance matrix computed in the standard error estimation step is (nearly) computationally singular then that estimate is considered non-converged, possibly due to model degeneracy.

For empirical network estimations where there are not a large number of automated estimations to process, visual inspection of the parameter and statistic trace plots is used as a heuristic to confirm convergence. In the case of the simulated networks where large numbers of estimations are run, we automate the additional heuristic that estimations with numeric overflow (“NaN” values) or “huge” (greater in magnitude than 10^10^) parameter values are non-converged.

An additional heuristic (visual) convergence test for modeling empirical networks is to plot various network statistics of the observed network on the same plot as the distribution of these statistics in the EE algorithm simulated networks. This is the same principle as the t-ratio test, but it includes statistics other than those explicitly included in the model in order to give some indication of how well the model fits. The statistics included are the degree distribution, reciprocity, giant component size, average local and global clustering coefficients, triad census, geodesic distribution, and edgewise and dyadwise shared partners (similar to goodness-of-fit plots in statnet [30–33]).

Note, however, that these plots are not goodness-of-fit plots, as the simulated networks are not generated *ab initio* (e.g. from the empty network) from the estimated parameters, but rather are the networks that have been simulated in the EE algorithm where the starting point is the observed network. Hence the plots may be “over-optimistic” in indicating a good model fit: however a plot clearly showing a poor model fit definitely indicates lack of convergence or a poor model.

In addition, for every large networks it is impractical to compute some distributions in reasonable time (such as the edgewise and dyadwise shared partners, and the geodesic distribution) and so these plots are excluded for very large networks. An example of a convergence plot is shown in S1 Fig.

## Results and discussion

### Simulated networks

**Fig 7:**
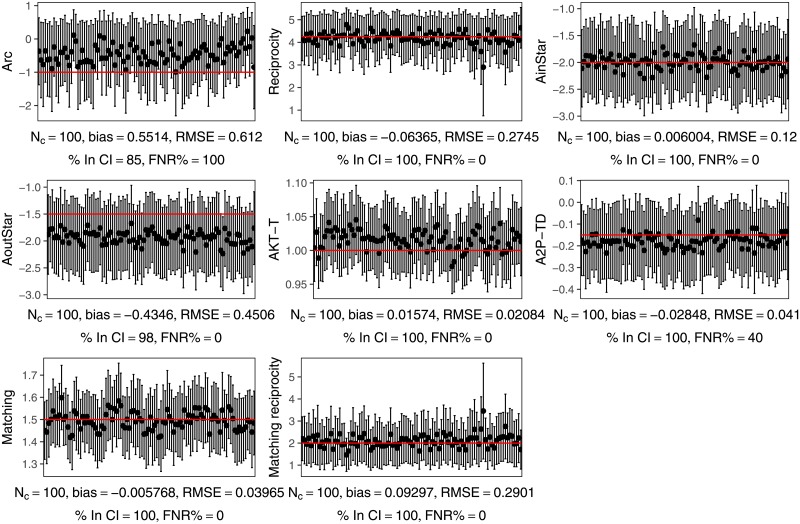
EstimNetDirected parameter estimates for 2000 node networks with categorical attribute. The error bars show the nominal 95% confidence interval. The horizontal line shows the true value of the parameter, and each plot is also annotated with the mean bias, root mean square error (RMSE), the percentage of samples for which the true value is inside the confidence interval, the coverage (% in CI), and the Type II error rate (False Negative Rate, FNR). N_C_ is the number of networks (of the total 100) for which a converged estimate was found, each of which is shown on the plot.

**Fig 8:**
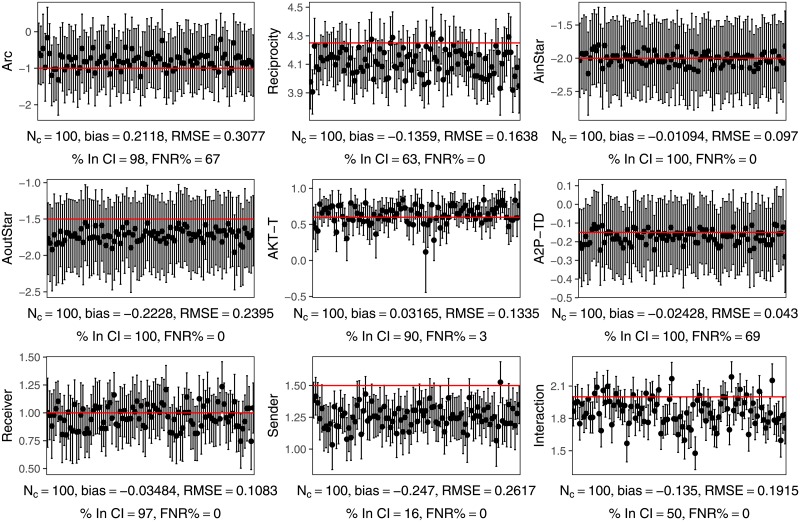
EstimNetDirected parameter estimates for 2000 node networks with binary attribute. The error bars show the nominal 95% confidence interval. The horizontal line shows the true value of the parameter, and each plot is also annotated with the mean bias, root mean square error (RMSE), the percentage of samples for which the true value is inside the confidence interval, the coverage (% in CI), and the Type II error rate (False Negative Rate, FNR). N_C_ is the number of networks (of the total 100) for which a converged estimate was found, each of which is shown on the plot.

Table 2 shows the bias, RMSE, Type II error rate, and coverage in estimating the simulated networks. It can be seen that the Type II error rate in inference is very low on all parameters except for Arc and and A2P-TD. In the case of the Arc parameter, this is of little concern as we do not in practice need to make a statistical inference on this parameter, as previously noted it is analogous to an intercept and simply used to control for network density. Figs 7 and 8, plotting the point estimates and their error bars, shed some more light on this problem. It seems the high Type II error rate for Arc and A2P-TD is at least partly explained by the small magnitude of the true values of these parameters. The coverage in most of these cases indicates overly conservative error estimates (higher than the nominal 95%), and the Type II error rate is high.

**Table 2:** Results from estimation of simulated networks using EstimNetDirected estimating Type II error rate.

N	Attributes	Effect	Bias	RMSE	estim.	lower	upper	in C.I. (%)	*N*_*C*_	$\bar{N_{R}}$
2000	Categorical	A2P-TD	-0.0285	0.0411	40	31	50	100	100	2.00
2000	Categorical	AinS	0.0060	0.1298	0	0	4	100	100	2.00
2000	Categorical	AKT-T	0.0157	0.0208	0	0	4	100	100	2.00
2000	Categorical	AoutS	-0.4346	0.4506	0	0	4	98	100	2.00
2000	Categorical	Arc	0.5514	0.6120	100	96	100	85	100	2.00
2000	Categorical	Matching	-0.0058	0.0396	0	0	4	100	100	2.00
2000	Categorical	MatchingReciprocity	0.0930	0.2901	0	0	4	100	100	2.00
2000	Categorical	Reciprocity	-0.0636	0.2745	0	0	4	100	100	2.00
2000	Binary	A2P-TD	-0.0243	0.0439	69	59	77	100	100	1.98
2000	Binary	AinS	-0.0109	0.0974	0	0	4	100	100	1.98
2000	Binary	AKT-T	0.0316	0.1335	3	1	8	90	100	1.98
2000	Binary	AoutS	-0.2228	0.2395	0	0	4	100	100	1.98
2000	Binary	Arc	0.2118	0.3077	67	57	75	98	100	1.98
2000	Binary	Interaction	-0.1350	0.1915	0	0	4	50	100	1.98
2000	Binary	Receiver	-0.0348	0.1083	0	0	4	97	100	1.98
2000	Binary	Reciprocity	-0.1359	0.1638	0	0	4	63	100	1.98
2000	Binary	Sender	-0.2470	0.2617	0	0	4	16	100	1.98

The “estim.”, “lower”, and “upper” columns show the point estimate and lower and upper 95% confidence interval (C.I.), respectively, of the Type II error rate (false negative rate). This C.I. is computed as the Wilson score interval. The “in C.I. (%)” column is the coverage rate for the nominal 95% confidence interval of the EstimNetDirected point and standard error estimates. Results are over 100 networks, each of which has 32 parallel estimation runs. *N*_*C*_ is the number of networks for which a converged estimate was found (out of 100). $\bar{N_{R}}$ is the mean number of runs that converged (out of 32). Runs that did not converge are not included in the estimates.

Additionally, in the case of the Arc parameter especially, the parameter estimates appear to be biased. Although the Type II error rate is very low, in the case of the networks with binary attribute, the coverage rate is low for the Sender, Interaction, and (to a lesser degree) Reciprocity parameters. Fig 8 shows that this appears to be because of bias in these parameter estimates. In the case of the networks with a categorical attribute, Fig 7 shows positive bias in the Arc parameter resulting in a high Type II error rate despite lower than nominal coverage.

We should not be surprised by bias in the parameter estimates, as the MLE for ERGM canonical parameters is biased precisely because it is unbiased by construction in the mean value parameter space [61] (that is, the mean values of the statistics, not the corresponding ERGM parameters). For this reason, van Duijn, Gile, & Handcock [61] propose a framework for assessing estimators in which bias is compared in the mean value parameter space, by generating large numbers of simulated networks from the estimated parameter values, and comparing them to the statistics of the original simulated networks. However for large directed networks this procedure is impractical due to the time it takes to simulate each set of networks (the very reason the EE algorithm is so fast is that it avoids doing this). In addition, as the purpose of ERGM parameter estimation is usually to make statistical inferences from the estimated parameters, it is useful to measure the inferential error in this procedure.

In Tables 2 and 3, the 95% confidence interval for the Type II and Type I error rate, respectively, is computed as the Wilson score interval [62].

**Table 3:** Results from estimation of full network using EstimNetDirected estimating Type I error rate.

N	Attributes	Effect	Bias	RMSE	estim.	lower	upper	in C.I. (%)	*N*_*C*_	$\bar{N_{R}}$
2000	Categorical	A2P-TD	-0.0217	0.0657	1	0	5	99	100	1.94
2000	Categorical	AinS	-0.0017	0.0648	1	0	5	99	100	2.00
2000	Categorical	AKT-T	-0.0154	0.0837	0	0	4	100	100	2.00
2000	Categorical	AoutS	-0.0129	0.0706	1	0	5	99	100	2.00
2000	Categorical	Matching	0.0239	0.0440	11	6	19	89	100	2.00
2000	Categorical	MatchingReciprocity	0.1246	0.1981	9	5	16	91	100	2.00
2000	Categorical	Reciprocity	0.4809	0.5493	2	1	7	98	100	0.86
2000	Binary	A2P-TD	-0.0143	0.0198	2	1	7	98	100	2.00
2000	Binary	AinS	-0.1234	0.1830	1	0	5	99	100	2.00
2000	Binary	AKT-T	-0.2473	0.5563	1	0	5	99	100	9.32
2000	Binary	AoutS	-0.0011	0.0954	0	0	4	100	100	2.00
2000	Binary	Interaction	-0.7966	3.0590	4	1	15	96	46	7.02
2000	Binary	Receiver	0.0313	0.1577	5	2	11	95	100	1.33
2000	Binary	Reciprocity	-0.3127	1.2360	0	0	14	100	24	6.96
2000	Binary	Sender	0.0244	0.1252	2	1	7	98	100	0.73

The “estim.”, “lower”, and “upper” columns show the point estimate and lower and upper 95% confidence interval (C.I.), respectively, of the Type I error rate (false positive rate). This C.I. is computed as the Wilson score interval. The “in C.I. (%)” column is the coverage rate for the nominal 95% confidence interval of the EstimNetDirected point and standard error estimates. Results are over 100 networks, each of which has 32 parallel estimation runs. *N*_*C*_ is the number of networks for which a converged estimate was found (out of 100). $\bar{N_{R}}$ is the mean number of runs that converged (out of 32). Runs that did not converge are not included in the estimates.

Table 3 shows the coverage and Type I error rates estimated from simulated networks with zero effects. Note that in this case, coverage and Type I error rate are effectively the same thing (as percentages, coverage is 100 − *α* where *α* is the Type I error rate) as the known true value of the parameter is zero by design. This table shows that the Type I error rate in all but two cases is within the nominal 5% range. In one case, Matching Reciprocity for the categorical networks, the point estimate of the Type I error rate is 9% but the 95% confidence interval extends down to 5%. However the other case, Matching for categorical networks, the point estimate of the Type I error rate is 11% and the confidence interval extends down to only 6%, so for this particular case the Type I error rate is too high. We note that the coverage for this zero parameter is only 89%, so it would appear that our method is potentially subject to inferential error in such cases where Matching Reciprocity is included in the model but the corresponding baseline Matching parameter is not. We would recommend not using such a model, and always including the corresponding baseline parameters, as is standard practice in ERGM model building, where configurations are nested within one another (Ch. 3 of [1]).

Also note that for two of the sets of simulated networks, the binary node attribute networks with the Interaction or Reciprocity effects set to zero, the number of runs and networks for which estimations converged is very low (less than half). This is not problematic, as these tests, where an effect not present in the network is included in the model, could be considered instances of model mis-specification, so the possibility of estimations not converging is to be expected. Note that for estimations shown in Table 2, where the model is exactly correct (it is the same model that generated the networks), converged estimates are obtained for all the simulated networks.

### Empirical network example

Table 4 shows a model estimated for the Pokec online social network with the 20 highest degree hubs removed (*N* = 1 632 783). This estimation took approximately 22 hours on cluster nodes with Intel Xeon E5-2650 v3 2.30GHz processors using two parallel tasks with 512 GB RAM each.

**Table 4:** Parameter estimates for the Pokec online social network with hubs removed.

Effect	Estimate	Std. error	
Arc	-20.642	0.381	*
Isolates	-7.711	2.269	*
Reciprocity	33.473	3.834	*
Popularity spread (AinS)	1.720	0.128	*
Activity spread (AoutS)	1.900	0.280	*
Two-path (A2P-T)	-0.014	0.003	*
Shared popularity (A2P-D)	0.021	0.003	*
Shared activity (A2P-U)	0.022	0.003	*
Path closure (AKT-T)	2.151	0.411	*
Popularity closure (AKT-D)	2.270	0.439	*
Activity closure (AKT-U)	2.270	0.407	*
Sender age	0.021	0.014	
Receiver age	0.022	0.013	
Diff age	-0.099	0.012	*
Matching gender	-1.164	0.287	*
Matching region	3.172	0.543	*

Asterisks (*) indicate statistical significance at *p* < 0.05.

Regarding structural features, these results confirm centralization on both in- and out-degree in the Pokec online social network. There is a significantly positive reciprocity effect: friendships are more likely to be reciprocated than not, conditional on the other features in the model. There is also positive activity closure: people who send friendship ties to the same people also tend to be friends. There is also positive path (transitive) closure, in combination with negative two-paths: friends of friends tend also to be friends. This can also be interpreted in terms of the “forbidden triad” [63], in which an open two-path (of what we assume to be “strong” ties as they represent friendship) is not closed transitively. Such a triad is indeed less likely than by chance, conditional on the other parameters in the model, in this network.

There is homophily on both region and age: people who live in the same region are more likely to be friends than those who live in different regions, and people of similar ages are more likely to be friends. Interestingly, there is significant heterophily on the gender attribute: people of different genders are more likely to be friends on this online social network.

The convergence test plot for the Pokec online social network estimation is shown in S1 Fig.

The algorithm parameters used for this estimation are shown in Table A in S1 Appendix in the column for the Pokec network. As a general guideline, we recommend using these parameters, with the exception of EEsteps. It may be useful to start with the default value of 1 000 (or even smaller) for this parameter, to relatively quickly obtain initial results and check the trace plots for any obvious failure to converge, such as a parameter value clearly diverging (due to a bad model for instance). If there are no obvious problems then the EEsteps parameter can be increased if the t-ratios or trace plots (as described in the section “Convergence tests”) indicate that the estimation has not yet converged.

## Conclusion

We have demonstrated an implementation of the EE algorithm for ERGM parameter estimation capable of estimating models for social networks with over one million nodes, which is far larger than previously possible (without using network sampling). However there are several limitations and scope for future work. The implementation described here requires tuning some algorithm parameters (Table A in S1 Appendix), however a simplified version of the EE algorithm requires fewer parameters [25] and may make it easier to obtain converged models.

Although the use of hash tables to efficiently store the sparse two-path matrices allows scalability to networks of millions of nodes, it depends on sufficient sparsity of these matrices, and not all empirical networks of interest satisfy this requirement. For example the physician referral network described by An et al. [64], although having approximately one million nodes, making it smaller than the Pokec online social network, does not have a sufficiently sparse two-path table for our implementation to work even with the largest memory cluster node available to us (512 GB memory). Further work is required to find a means of alleviating this problem.

Although the convergence heuristic plots we have described resemble a goodness-of-fit test superficially, they are not actually goodness-of-fit tests. The difficulty of generating (simulating) numbers of very large networks from estimated ERGM parameters for conventional goodness-of-fit tests for ERGM makes these methods impractical for such large networks. One possibility to investigate is to simulate snowball samples from the estimated parameters and compare the distribution of network statistics of these simulated samples to the corresponding distributions of network statistics in snowball sample taken from the observed network. More generally, the idea of such goodness-of-fit tests for extremely large networks may have to be fundamentally re-examined, in light of the difficulty of any simple model adequately fitting such a large network—and the homogeneity assumption that the same local processes operate across such a large network may also no longer be realistic.

In addition, the ERGM MLE bias in canonical parameters may need to be addressed by some bias correction technique, as was originally explored for maximum pseudolikelihood estimation [61], but does not appear to have been successfully pursued since for other, now preferred (not least due to the results of [61]), estimation methods.

An important next step is the strengthening of the theoretical basis for the the EE algorithm. Existing commonly used methods for ERGM parameter estimation are based on well-known algorithms with well developed theoretical foundations such as stochastic approximation [5] using the Robbins–Monro algorithm [65] or MCMCMLE [6] based on the Geyer–Thompson method [66] with increasingly sophisticated variants [9]. In contrast, there are no theoretical guarantees behind the EE algorithm [12], and although contrastive divergence [39, 40] and persistent contrastive divergence [36, 37] are becoming widely used, more theoretical work is required to understand their convergence properties in general, and for ERGM parameter estimation in particular [67]. Regarding the EE algorithm, the experiments with simulated networks described in this paper give encouragement for the validity and usefulness of this approach, but further work to understand its convergence properties is a potentially fruitful area of research [25].

## Supporting information

S1 Appendix Algorithm descriptions and EstimNetDirected parameter settings. (PDF)

S1 Fig Convergence test plot for Pokec (hubs removed) estimation. The observed network statistics are plotted in red with the statistics of the EE algorithm simulated networks on the same plot as black boxplots, or blue on histogram plots. Note that on the triad census plots, triads 003, 012, and 102 are omitted as the extremely large counts cause numeric overflow in the igraph library [50] for a network this large.
(PDF)
