# Correction: Exponential random graph model parameter estimation for very large directed networks

**DOI:** 10.1371/journal.pone.0231023

**Published:** 2020-03-24

**Authors:** The PLOS ONE Staff

There are errors in the entries in the final columns of Tables 2 and 3 introduced during the typesetting process. The publisher apologizes for these errors. Please see the correct Tables 2 and 3 here.

**Table 2:** Results from estimation of simulated networks using EstimNetDirected estimating Type II error rate.

N	Attributes	Effect	Bias	RMSE	estim.	lower	upper	in C.I. (%)	*N*_*C*_	$\bar{N_{R}}$
2000	Categorical	A2P-TD	-0.0285	0.0411	40	31	50	100	100	32.00
2000	Categorical	AinS	0.0060	0.1298	0	0	4	100	100	32.00
2000	Categorical	AKT-T	0.0157	0.0208	0	0	4	100	100	32.00
2000	Categorical	AoutS	-0.4346	0.4506	0	0	4	98	100	32.00
2000	Categorical	Arc	0.5514	0.6120	100	96	100	85	100	32.00
2000	Categorical	Matching	-0.0058	0.0396	0	0	4	100	100	32.00
2000	Categorical	MatchingReciprocity	0.0930	0.2901	0	0	4	100	100	32.00
2000	Categorical	Reciprocity	-0.0636	0.2745	0	0	4	100	100	32.00
2000	Binary	A2P-TD	-0.0243	0.0439	69	59	77	100	100	31.98
2000	Binary	AinS	-0.0109	0.0974	0	0	4	100	100	31.98
2000	Binary	AKT-T	0.0316	0.1335	3	1	8	90	100	31.98
2000	Binary	AoutS	-0.2228	0.2395	0	0	4	100	100	31.98
2000	Binary	Arc	0.2118	0.3077	67	57	75	98	100	31.98
2000	Binary	Interaction	-0.1350	0.1915	0	0	4	50	100	31.98
2000	Binary	Receiver	-0.0348	0.1083	0	0	4	97	100	31.98
2000	Binary	Reciprocity	-0.1359	0.1638	0	0	4	63	100	31.98
2000	Binary	Sender	-0.2470	0.2617	0	0	4	16	100	31.98

The “estim.”, “lower”, and “upper” columns show the point estimate and lower and upper 95% confidence interval (C.I.), respectively, of the Type II error rate (false negative rate). This C.I. is computed as the Wilson score interval. The “in C.I. (%)” column is the coverage rate for the nominal 95% confidence interval of the EstimNetDirected point and standard error estimates. Results are over 100 networks, each of which has 32 parallel estimation runs. *N*_*C*_ is the number of networks for which a converged estimate was found (out of 100). $\bar{N_{R}}$ is the mean number of runs that converged (out of 32). Runs that did not converge are not included in the estimates.

**Table 3:** Results from estimation of full network using EstimNetDirected estimating Type I error rate.

N	Attributes	Effect	Bias	RMSE	estim.	lower	upper	in C.I. (%)	*N*_*C*_	$\bar{N_{R}}$
2000	Categorical	A2P-TD	-0.0217	0.0657	1	0	5	99	100	31.94
2000	Categorical	AinS	-0.0017	0.0648	1	0	5	99	100	32.00
2000	Categorical	AKT-T	-0.0154	0.0837	0	0	4	100	100	32.00
2000	Categorical	AoutS	-0.0129	0.0706	1	0	5	99	100	32.00
2000	Categorical	Matching	0.0239	0.0440	11	6	19	89	100	32.00
2000	Categorical	MatchingReciprocity	0.1246	0.1981	9	5	16	91	100	32.00
2000	Categorical	Reciprocity	0.4809	0.5493	2	1	7	98	100	30.86
2000	Binary	A2P-TD	-0.0143	0.0198	2	1	7	98	100	32.00
2000	Binary	AinS	-0.1234	0.1830	1	0	5	99	100	32.00
2000	Binary	AKT-T	-0.2473	0.5563	1	0	5	99	100	29.32
2000	Binary	AoutS	-0.0011	0.0954	0	0	4	100	100	32.00
2000	Binary	Interaction	-0.7966	3.0590	4	1	15	96	46	7.02
2000	Binary	Receiver	0.0313	0.1577	5	2	11	95	100	31.33
2000	Binary	Reciprocity	-0.3127	1.2360	0	0	14	100	24	6.96
2000	Binary	Sender	0.0244	0.1252	2	1	7	98	100	30.73

The “estim.”, “lower”, and “upper” columns show the point estimate and lower and upper 95% confidence interval (C.I.), respectively, of the Type I error rate (false positive rate). This C.I. is computed as the Wilson score interval. The “in C.I. (%)” column is the coverage rate for the nominal 95% confidence interval of the EstimNetDirected point and standard error estimates. Results are over 100 networks, each of which has 32 parallel estimation runs. *N*_*C*_ is the number of networks for which a converged estimate was found (out of 100). $\bar{N_{R}}$ is the mean number of runs that converged (out of 32). Runs that did not converge are not included in the estimates.
