# Supplementary material for: Exponential random graph model parameter estimation for very large directed networks
Source: PLoS One. 2020 Jan 24;15(1):e0227804. doi: 10.1371/journal.pone.0227804 (PMC6980401; doi:10.1371/journal.pone.0227804)
Supplement: S1 Appendix — (PDF) [file pone.0227804.s001.pdf]

Pseudocode for the EE algorithm is detailed below. This algorithm uses an ERGM sampler described immediately following it, the IFD sampler [1], however other ERGM samplers may be used, and in particular the "basic" sampler [2, 3], the pseudocode for which is detailed in the Supplementary Information of [4] was used for the simulated network estimations.

In the algorithm descriptions, vectors such as $\boldsymbol{\theta}$, $\boldsymbol{z}$, and $\boldsymbol{dz}$ have dimension equal to the number of model parameters, s. All vector operations are elementwise, e.g. $\boldsymbol{dz}^2$ is the vector consisting of the square of each element of $\boldsymbol{dz}$ and $\boldsymbol{D} \odot \boldsymbol{dz}$ is the elementwise (Hadamard) product of $\boldsymbol{D}$ and $\boldsymbol{dz}$ (a vector of the same dimension, s, as both $\boldsymbol{D}$ and $\boldsymbol{dz}$).

Precondition: x_{obs} is the observed graph, $\boldsymbol{\theta_0}$ is the initial parameter estimate, $\boldsymbol{D_0}$ is the initial derivative estimate.

Postcondition: Returned value $\boldsymbol{\theta_t}$ is the estimated parameter value.

function EE($x_{\text{obs}}, \boldsymbol{\theta_0}, \boldsymbol{D_0}$)
  $K_A \leftarrow 10^{-4}$ ▷ Multiplier of $\boldsymbol{D}$ to get step size multiplier
  $c_1 \leftarrow 10^{-2}$ ▷ Minimum magnitude of $|\bar{\boldsymbol{\theta}}|$ (small positive constant)
  $c_2 \leftarrow 10^{-4}$ ▷ Multiplier of $|\bar{\boldsymbol{\theta}}|/\text{sd}\,(\boldsymbol{\theta})$ to limit $\boldsymbol{\theta}$ variance
  $K_{\text{IFD}} \leftarrow 0.1$ ▷ IFD sampler auxiliary parameter step size multiplier
  $V \leftarrow 0$ ▷ IFD sampler auxiliary parameter
  $M_{\text{outer}} \leftarrow 1000$ ▷ Steps of Algorithm EE
  $M_{\text{inner}} \leftarrow 100$ ▷ Inner iterations of Algorithm EE
  $m \leftarrow 1000$ ▷ Number of sampler iterations
  $t \leftarrow 0$
  $x \leftarrow x_{\text{obs}}$
  $\boldsymbol{D} \leftarrow \boldsymbol{D_0}$
  $\boldsymbol{dz} \leftarrow \boldsymbol{0}$ ▷ Vector of accumulated change statistics
  for $i \leftarrow 1$ to M_{outer} **do**
    for $j \leftarrow 1$ to M_{inner} **do**
      $(\boldsymbol{dzAdd}, \boldsymbol{dzDel}) \leftarrow \text{SAMPLER}(x, \boldsymbol{\theta_t}, m, K_{\text{IFD}}, V)$
      $\boldsymbol{dz} \leftarrow \boldsymbol{dz} + \boldsymbol{dzAdd} - \boldsymbol{dzDel}$ ▷ Accumulate accepted change statistics
      $\boldsymbol{\theta_{t+1}} \leftarrow \boldsymbol{\theta_t} - \text{sign}(\boldsymbol{dz}) \odot K_A \boldsymbol{D} \odot \boldsymbol{dz}^2$
      $t \leftarrow t + 1$
    end for
    $\boldsymbol{D} \leftarrow \boldsymbol{D} \odot \left[c_2 \frac{\max\left(|\overline{\boldsymbol{\theta_{t-M_{\text{inner}} \leq k < t}}}|, c_1\right)}{\text{sd}\left(\boldsymbol{\theta_{t-M_{\text{inner}} \leq k < t}}\right)} \right]^{\frac{1}{2}}$
  end for
  return $\boldsymbol{\theta_t}$
end function

The algorithm to sample from ERGM distributions with Metropolis–Hastings using the IFD sampler [1] is described below. Note that the Arc parameter θ_L must not be included in the model when using the IFD sampler, instead it is calculated from the IFD sampler auxiliary parameter V as

$$\theta_L = V - \log\left(\frac{L_{\max} - L_{\text{obs}}}{L_{\text{obs}} + 1}\right)$$

where $L_{\max} = N(N-1)$ is the number of possible arcs in a directed graph and L_{obs} is the number of arcs in the observed graph x_{obs}.

Precondition: x is a directed graph, $\boldsymbol{\theta}$ is vector of parameters, m is number of sampler iterations, K_{IFD} is the multiplier for the auxiliary parameter step size, e.g. $K_{\text{IFD}} = 0.1$. Initially set IFD auxiliary parameter $V = 0$.

Postcondition: Return value $(\boldsymbol{dzAdd}, \boldsymbol{dzDel})$ accumulated change statistics of accepted (add, delete) moves. The graph x is updated by the accepted moves and the IFD auxiliary parameter V is updated.

function SAMPLER($x, \boldsymbol{\theta}, m, K_{\text{IFD}}, V$)
  isDelete $\leftarrow$ False
  $N_{\text{add}} \leftarrow 0$ ▷ number of add moves
  $N_{\text{del}} \leftarrow 0$ ▷ number of delete moves
  $\boldsymbol{dzAdd} \leftarrow \mathbf{0}$
  $\boldsymbol{dzDel} \leftarrow \mathbf{0}$
  for $w \leftarrow 1$ to m **do**
    if isDelete **then** ▷ Delete move
      $N_{\text{del}} \leftarrow N_{\text{del}} + 1$
      Choose two nodes i, j $(i \neq j)$ with arc $i \rightarrow j$ uniformly at random
    else ▷ Add move
      $N_{\text{add}} \leftarrow N_{\text{add}} + 1$
      Choose two nodes i, j $(i \neq j)$ with no arc $i \rightarrow j$ uniformly at random
    end if
    Compute change statistic dz_A for adding arc $i \rightarrow j$ (if $\neg$ isDelete) or deleting arc $i \rightarrow j$ (if isDelete) for each statistic A
    $V_s \leftarrow -1$ if isDelete else 1
    $\alpha \leftarrow \min\left\{1, \exp\left(\sum_A \left[\theta_A dz_A\right] + V_s V\right)\right\}$ ▷ proposal acceptance probability
    if $\text{Unif}(0,1) < \alpha$ **then** ▷ Accept change with probability α
      if isDelete **then**
        $\boldsymbol{dzDel} \leftarrow \boldsymbol{dzDel} - \boldsymbol{dz}$
        $x_{ij} \leftarrow 0$
      else
        $\boldsymbol{dzAdd} \leftarrow \boldsymbol{dzAdd} + \boldsymbol{dz}$
        $x_{ij} \leftarrow 1$
      end if
      isDelete $\leftarrow \neg$ isDelete
    end if
  end for
  $V_{\text{step}} \leftarrow \frac{(N_{\text{del}} - N_{\text{add}})^2}{(N_{\text{del}} + N_{\text{add}})^2}$
  if $N_{\text{del}} - N_{\text{add}} > 0$ **then**
    $V \leftarrow V - K_{\text{IFD}} V_{\text{step}}$
  else
    $V \leftarrow V + K_{\text{IFD}} V_{\text{step}}$
  end if
  if $\frac{|N_{\text{del}} - N_{\text{add}}|}{N_{\text{del}} + N_{\text{add}}} > 0.8$ **then**
    Warn that K_{IFD} might be too small.
  end if
  return $(\boldsymbol{dzAdd}, \boldsymbol{dzDel})$
end function

Table A. EstimNetDirected parameter settings.

Parameter name	Pseudocode	Simulated	Pokec
ACA_S	$K1_A$	0.1	0.1
ACA_EE	K_A	10^{-9}	10^{-7}
compC	c_2	0.01	0.01
samplerSteps	m	1000	1000
Ssteps	$M1$	50	1000
EEsteps	M_{outer}	500	1500
EinnerSteps	M_{inner}	100	100
useIFDsampler	—	False	True
ifd_K	K_{IFD}	—	0.1

"Pseudocode" is the notation used for the parameter in this Appendix or Supplementary Information of [4]. "Simulated" is the value used for estimating the simulated networks and "Pokec" is the value used in estimating the model for the Pokec online social network.

References

[1] Byshkin M, Stivala A, Mira A, Krause R, Robins G, Lomi A. Auxiliary parameter MCMC for exponential random graph models. J Stat Phys. 2016;165(4):740–754.

[2] Snijders TAB. Markov chain Monte Carlo estimation of exponential random graph models. J Soc Struct. 2002;3(2):1–40.

[3] Wang P, Robins G, Pattison P. PNet: program for the simulation and estimation of exponential random graph (p^*) models; 2009.

[4] Byshkin M, Stivala A, Mira A, Robins G, Lomi A. Fast maximum likelihood estimation via Equilibrium Expectation for large network data. Sci Rep. 2018;8:11509.
