# Supplementary figures and images for: Exponential random graph model parameter estimation for very large directed networks
Source: PLoS One. 2020 Jan 24;15(1):e0227804. doi: 10.1371/journal.pone.0227804 (PMC6980401; doi:10.1371/journal.pone.0227804)

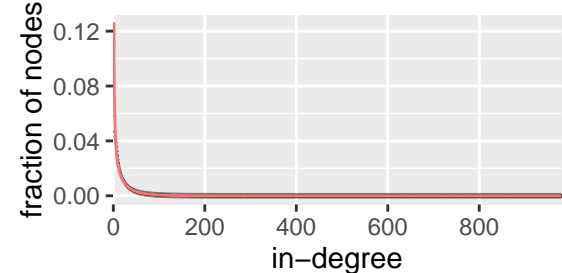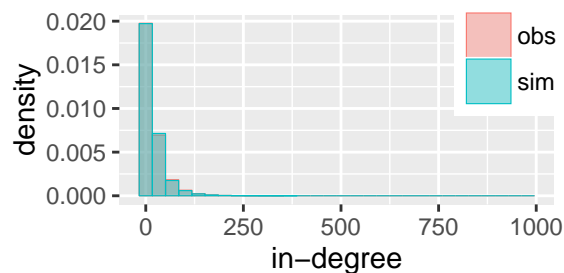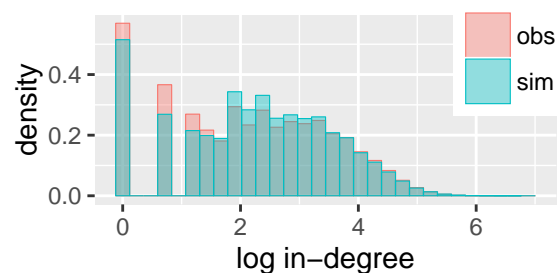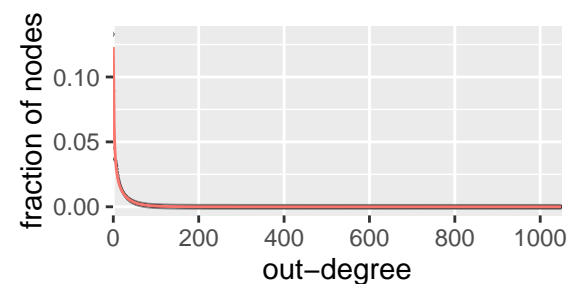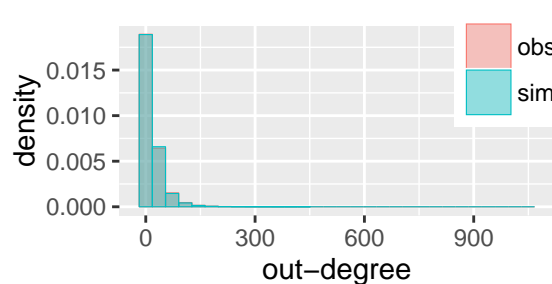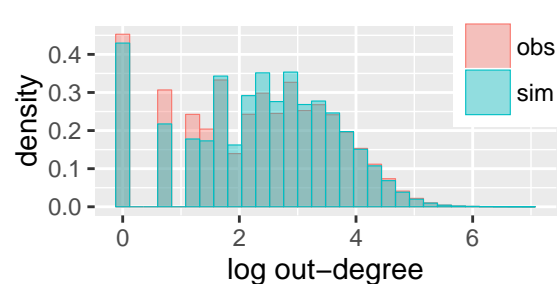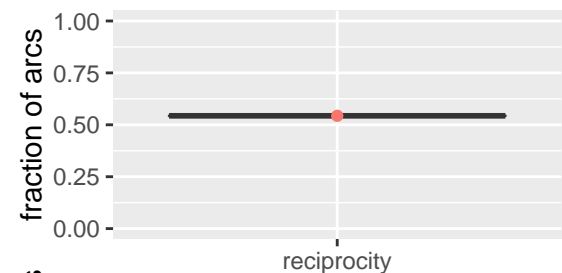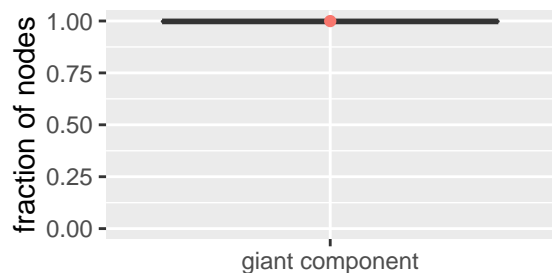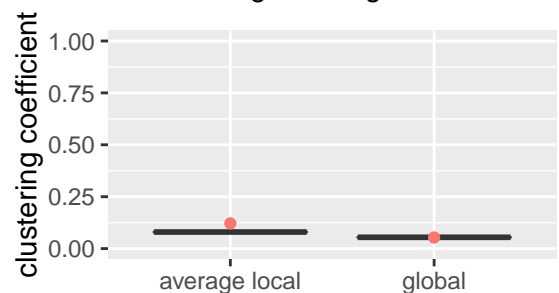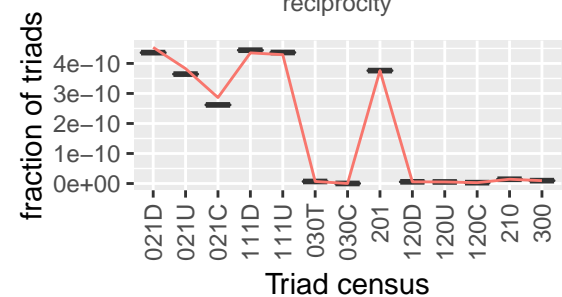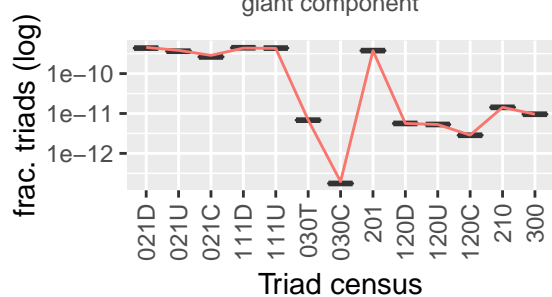

Supplement: S1 Fig — The observed network statistics are plotted in red with the statistics of the EE algorithm simulated networks on the same plot as black boxplots, or blue on histogram plots. Note that on the triad census plots, triads 003, 012, and 102 are omitted as the extremely large counts cause numeric overflow in the igraph library [50] for a network this large. (PDF) [file pone.0227804.s002.pdf]
